# iLIR: A web resource for prediction of Atg8-family interacting proteins

**DOI:** 10.4161/auto.28260

**Published:** 2014-02-26

**Authors:** Ioanna Kalvari, Stelios Tsompanis, Nitha C Mulakkal, Richard Osgood, Terje Johansen, Ioannis P Nezis, Vasilis J Promponas

**Affiliations:** 1 Bioinformatics Research Laboratory; Department of Biological Sciences; University of Cyprus; Nicosia, Cyprus; 2 Student in International Erasmus Exchange Programme (2012–2013); 3 School of Life Sciences; University of Warwick; Coventry, UK; 4 Molecular Cancer Research Group; Institute of Medical Biology; University of Tromsø; Tromsø, Norway

**Keywords:** macroautophagy, selective autophagy, LC3 interacting region-motif, Atg8-family interacting proteins, web server, prediction

## Abstract

Macroautophagy was initially considered to be a nonselective process for bulk breakdown of cytosolic material. However, recent evidence points toward a selective mode of autophagy mediated by the so-called selective autophagy receptors (SARs). SARs act by recognizing and sorting diverse cargo substrates (e.g., proteins, organelles, pathogens) to the autophagic machinery. Known SARs are characterized by a short linear sequence motif (LIR-, LRS-, or AIM-motif) responsible for the interaction between SARs and proteins of the Atg8 family. Interestingly, many LIR-containing proteins (LIRCPs) are also involved in autophagosome formation and maturation and a few of them in regulating signaling pathways. Despite recent research efforts to experimentally identify LIRCPs, only a few dozen of this class of—often unrelated—proteins have been characterized so far using tedious cell biological, biochemical, and crystallographic approaches. The availability of an ever-increasing number of complete eukaryotic genomes provides a grand challenge for characterizing novel LIRCPs throughout the eukaryotes. Along these lines, we developed iLIR, a freely available web resource, which provides in silico tools for assisting the identification of novel LIRCPs. Given an amino acid sequence as input, iLIR searches for instances of short sequences compliant with a refined sensitive regular expression pattern of the extended LIR motif (xLIR-motif) and retrieves characterized protein domains from the SMART database for the query. Additionally, iLIR scores xLIRs against a custom position-specific scoring matrix (PSSM) and identifies potentially disordered subsequences with protein interaction potential overlapping with detected xLIR-motifs. Here we demonstrate that proteins satisfying these criteria make good LIRCP candidates for further experimental verification. Domain architecture is displayed in an informative graphic, and detailed results are also available in tabular form. We anticipate that iLIR will assist with elucidating the full complement of LIRCPs in eukaryotes.

## 1. Introduction

Macroautophagy is a highly regulated catabolic process conserved throughout the eukaryotes, during which damaged or excessive cellular components are recycled.^1^ Autophagy starts with the nucleation of a double-membrane sequestering compartment (phagophore), which elongates and eventually closes, thus isolating its contents from the cytoplasm and forming an autophagosome.^2^ Autophagosomes go through a maturation process, which involves fusion with endosomes and/or lysosomes; their outer membrane fuses with that of the lysosome, followed by the degradation of their inner membrane and the sequestered content by acidic lysosomal hydrolases.^1^

Autophagy was initially considered a cellular response to starvation.^3^ Nowadays, it is considered as an essential homeostatic mechanism in eukaryotic cells, since it is responsible for the removal of excess or aggregated proteins or damaged organelles. Thus, autophagy plays an important role in the cellular mechanisms for quality control of proteins and organelles.^3^ Importantly, there is growing evidence that autophagy plays central roles in key processes, including differentiation (e.g., cell remodeling),^4^ development (e.g., embryogenesis),^4^^-^^6^ miRNA regulation,^7^ innate and adaptive immunity,^8^ and inflammation.^9^ Therefore, it is no surprise that recent findings indicate that deregulation of autophagy is related (often in complex ways) to several pathophysiological processes (among others: cancer, infectious diseases, metabolic, and neurodegenerative disorders) and aging.^10^ The complex interplay of autophagy with other cellular processes and external stimuli can be appreciated by the recently elucidated context-dependent roles of autophagy in cancer.^11^

Until very recently, the dominant view was that autophagy is a nonselective process for bulk degradation of cytosolic material. However, steadily accumulating evidence has highlighted the concept that sequestration and degradation of cytoplasmic material by autophagy can be conducted in a selective manner through receptor and adaptor proteins.^12^^,^^13^ Different types of cargos can be selected for autophagy, including proteins (often ubiquitinated), mitochondria (mitophagy),^14^ peroxisomes (pexophagy),^15^ nuclei (nucleophagy),^16^ lipid droplets (lipophagy),^17^ portions of the endoplasmic reticulum (reticulophagy),^18^ and even invading pathogens (xenophagy).^8^

### 1.1 The Atg8 family

A central position among autophagy-related (Atg) proteins belongs to the so-called autophagy-related 8 (Atg8) family, named after the prototype protein of the family, yeast Atg8. Structural data from homologs across the eukaryotes indicate that Atg8 family members adopt a β-grasp fold^19^^,^^20^ resembling a ubiquitin-like structure, despite no direct sequence similarities to ubiquitins.^21^ The yeast genome encodes a single member of the family, whereas family expansion is observed in higher eukaryotes (and a few protists).^22^ Members of the Atg8 family can be further classified based on sequence similarity into 3 subfamilies:^21^

• microtubule-associated protein 1 light chain 3 (MAP1LC3 or LC3), including LC3A, LC3B, LC3B2, LC3C;

• GABA(A) receptor-associated protein (GABARAP), including GABARAP, and GABARAPL1/GEC-1; and

• GABA(A) receptor-associated protein-like 2 (GABARAPL2/GATE-16/GEF2.

The complete functional repertoire of members of this family remains to be uncovered, especially in species with multiple paralogous genes.^22^ LC3 was originally characterized as the light chain of microtubule associated proteins 1A and 1B, whereas GABARAP and GABARAPL2 were first identified as intracellular vesicle trafficking factors.^23^ However, currently the main focus is on Atg8 homologs as important factors in autophagosome biogenesis. Atg8 conjugation to the phospholipid phosphatidylethanolamine (Atg8–PE) is a key event in the nucleation of the phagophore assembly site in yeast^24^ and in phagophore membrane elongation.^25^^,^^26^

### 1.2 Selective autophagy receptors

Selective autophagy is mediated by the so-called selective autophagy receptors, which act to recognize and tether the cargo substrate to the autophagic machinery for elimination. In their seminal work, Pankiv and colleagues^27^ used a combination of deletion mapping and point mutation techniques to trace the region of SQSTM1/p62 recognizing Atg8/LC3 down to a 22-residue peptide (coining the term LC3-interacting region [LIR]). Using information from SQSTM1 homologs, these authors could identify conserved residues/properties flanking an invariant tryptophan residue.

Atomic detail structural data for the mouse SQSTM1-LC3 interaction indicated that the actual interaction region is much shorter, mapping to a stretch of 11 conserved acidic and hydrophobic residues, the LC3 recognition sequence (LRS).^28^ Similar conclusions were derived when structural data became available for the human SQSTM1-LC3 interaction.^29^ Both structural works pinpointed the importance of a short tetrapeptide of the general form **W**XX**L** (X stands for any residue), with the tryptophan (W) and leucine (L) residues interacting with 2 distinct hydrophobic pockets of LC3, respectively.

In a follow-up survey, Noda and colleagues enhanced the description of this short motif to X_-3_X_-2_X_-1_**[WY]**X_1_X_2_**[LIV]**, where at least one of X_−3_X_−2_X_−1_, X_1_ and/or X_2_ are acidic residues and the square brackets enclose a group of alternative residue types which may occupy a single sequence site.^30^ Notably, position X_2_ of the motif is occupied by a glutamine residue (E) in yeast Atg19 and Atg34, the 2 cytoplasm-to-vacuole targeting (Cvt) pathway cargo receptors. They renamed the above motif as the Atg8-family interacting motif (AIM), based on the fact that the interactions observed in structures from yeast to human are quite similar.^30^

Recently, in an effort to identify the structural requirements of the assembly of the ULK (unc-51 like autophagy activating kinase) complex, Johansen and colleagues identified the LIR-motif in 3 LIRCPs (ULK1, ATG13, and RB1CC1/FIP200) using affinity isolation and peptide array overlay assays.^31^ In order to place their findings in a broader context, they collected 26 experimentally determined LC3 interacting regions and redefined the LIR-motif using a regular expression pattern: [DE][DEST][**WFY**][DELIV]X[**ILV**], where the positions marked in bold typeface correspond to the delimiters of the previously described **W**XX**L** motif.^31^

For the sake of simplicity, we hereafter collectively refer to the aforementioned short motifs as LIR-motifs. In particular, the regular expression pattern introduced in the paper by Alemu and colleagues^31^ will be referred to as the “canonical LIR-motif”, or cLIR-motif for short.

### 1.3 Computational approaches for identifying LIRCPs

Until now, no concerted effort to develop automated computational methods for large-scale identification of LIRCPs has been reported in the literature. This is probably due to:

• the relatively small number of experimentally validated LIRCPs/LIR-motifs on which predicting methods could be founded,

• the different LIR-motif definitions given in the literature,

• the notion that the LIR-motif itself does not guarantee interaction with proteins of the Atg8 family,^12^ and

• the lack of publicly available software tools of this type.

In fact, the patterns presented by Alemu and colleagues^31^ could be easily transformed to efficient software tools for identifying potential LIR-motifs in large numbers of sequences. However, these patterns suffer from a main drawback: since their authors generated these consensus representations mainly to explain their biological mode of action (i.e., Atg8 binding), these motifs are not sensitive enough, in the sense that they tend to miss a significant proportion of experimentally validated LIR-motifs (see also section 3.1).

To the best of our knowledge, the only approach applying more sophisticated sequence analysis methods for LIR detection, was the compilation of a position-specific scoring matrix (PSSM) using data from phage display screening of a randomized peptide library.^32^ These authors performed a PSSM-scan against the SwissProt database, which actually led to the discovery of the CALR/calreticulin LIR-motif and its interaction with GABARAP. Among other hits, this PSSM retrieved other known LIRCPs, namely CLTC (clathrin, heavy chain [Hc]) and BNIP3L/NIX.^32^ However, due to the narrow target of this PSSM and the fact that it is not available for public use, we conclude that, currently, the only way of computationally identifying potential LIRCPs is limited to performing sequence database searches looking for homologs of known LIRCPs.

## 2. Material and Methods

### 2.1 Sequence data

All protein sequences for LIRCPs used in this study were retrieved from the UniProt Knowledgebase (http://www.uniprot.org) by accession number (when available) or protein name and keywords and are listed in Table 1. UniProt entries were saved in text files both in SwissProt and FASTA formats. The particular entries used to derive the xLIR-motif and PSSM were manually checked for validity and for the actual position of LIR-motifs. Randomized versions of sequences were generated by shuffling (thus maintaining composition) using the shuffleseq program available from the EMBOSS explorer server (http://emboss.bioinformatics.nl/).

**Table:** **Table 1.** Sequences used in this study

		MOTIF							
**UNIPROT** **ID**	**UNIPROT** **ACC**	**Sequence**	**Position**	**Verified**	**cLIR**	**xLIR**	**Anchor**	**PSSM** **score** **(e-value)**	**Species**
**Data set from Alemu et al. 2012 **^31^									
ATG13_HUMAN	O75143	EGFQTV	166–171	No	No	Yes	No	11 (1.5e-01)	Human
		DDFVMI	442–447	Yes	Yes	Yes	Yes	20 (8.4e-03)	Human
Atg1_YEAST	P53104	REYVVV	427–432	Yes	No	Yes	Yes	14 (5.7e-02)	Yeast
Atg32_YEAST	P40458	GSWQAI	84–89	Yes	No	Yes	Yes	17 (2.2e-02)	Yeast
		KEYQSL	235–240	No	No	Yes	No	12 (1.1e-01)	Yeast
		LGYILL	524–529	No	No	Yes	No	10 (2.0e-01)	Yeast
ATG4B_HUMAN** [MM]	Q9Y4P1	LTYDTL	6–11	Yes	No	Yes	No	12 (1.1e-01)	Human
		PMFELV	347–352	No	No	Yes	No	10 (2.0e-01)	Human
		EDFEIL	386–391	No	Yes	Yes	No	17 (2.2e-02)	Human
Atg19_YEAST	P35193	LTWEEL	410–415	Yes	No	Yes	No	18 (1.6e-02)	Yeast
Atg3_YEAST	P40344	GDWEDL	268–273	Yes	No	Yes	No	22 (4.4e-03)	Yeast
BNI3L_HUMAN	O60238	SSWVEL	34–39	Yes	No	Yes	Yes	20 (8.4e-03)	Human
		AEFLKV	183–188	No	No	Yes	No	10 (2.0e-01)	Human
CALR_HUMAN	P27797	GGYVKL	107–112	No	No	Yes	No	12 (1.1e-01)	Human
		DEFTHL	166–171	No	No	Yes	No	14 (5.7e-02)	Human
		DDWDFL	198–203	Yes	Yes	Yes	Yes	26 (1.2e-03)	Human
CBL_HUMAN	P22681	DTYQHL	90–95	No	No	Yes	No	14 (5.7e-02)	Human
		LTYDEV	272–277	No	No	Yes	No	11 (1.5e-01)	Human
		FGWLSL	800–805	Yes	No	Yes	Yes	18 (1.6e-02)	Human
		REFVSI	893–898	No	No	Yes	Yes*	13 (7.9e-02)	Human
FUND1_HUMAN	Q8IVP5	DSYEVL	16–21	Yes	Yes	Yes	No	16 (3.0e-02)	Human
		GGFLLL	81–86	No	No	Yes	No	10 (2.0e-01)	Human
OPTN_HUMAN	Q96CV9	DSFVEI	176–181	Yes	Yes	Yes	Yes	15 (4.2e-02)	Human
Q8MQJ7_DROME	Q8MQJ7	ADYLSV	96–101	No	No	Yes	No	14 (5.7e-02)	*Drosophila*
		DDFVLV	389–394	Yes	Yes	Yes	Yes	17 (2.2e-02)	*Drosophila*
Q9SB64_ARATH	Q9SB64	RVWVLI	479–484	No	No	Yes	No	15 (4.2e-02)	*Arabidopsis*
		SEWDPI	659–664	Yes	No	Yes	No	20 (8.4e-03)	*Arabidopsis*
RBCC1_HUMAN	Q8TDY2	FDFETI	700–705	Yes	No	Yes	Yes	17 (2.2e-02)	Human
SQSTM_HUMAN** [LL]	Q13501	DDWTHL	336–341	Yes	No	Yes	Yes	24 (2.3e-03)	Human
STBD1_HUMAN** [LN]	O95210	EEWEMV	201–206	Yes	Yes	Yes	No	21 (6.1e-03)	Human
T53I1_HUMAN	Q96A56	DEWILV	29–34	Yes	Yes	Yes	Yes	20 (8.4e-03)	Human
TBC25_HUMAN	Q3MII6	EVYLSL	95–100	No	No	Yes	No	8 (3.9e-01)	Human
		EDWDII	134–139	Yes	Yes	Yes	No	24 (2.3e-03)	Human
TBCD5_HUMAN	Q92609	KEWEEL	57–62	Yes	No	Yes	No	20 (8.4e-03)	Human
		DDFILI	713–718	No	Yes	Yes	Yes*	17 (2.2e-02)	Human
		SGFTIV	785–790	Yes	No	Yes	Yes	11 (1.5e-01)	Human
T53I2_HUMAN	Q8IXH6	DGWLII	33–38	Yes	No	Yes	Yes	21 (6.1e-03)	Human
ULK1_HUMAN	O75385	DDFVMV	355–360	Yes	Yes	Yes	Yes	19 (1.2e-02)	Human
ULK2_HUMAN	Q8IYT8	DDFVLV	351–356	Yes	Yes	Yes	Yes	17 (2.2e-02)	Human
CLH1_HUMAN	Q00610	PDWIFL	512–517	Yes	No	Yes	No	22 (4.4e-03)	Human
		GMFTEL	1315–1320	No	No	Yes	No	11 (1.5e-01)	Human
		EDYQAL	1475–1480	No	No	Yes	No	16 (3.0e-02)	Human
DVL2_HUMAN	O14641	RMWLKI	442–447	Yes	No	Yes	No	18 (1.6e-02)	Human
FYCO1_HUMAN** [MM]	Q9BQS8	ADYQAL	644–649	No	No	Yes	Yes*	15 (4.2e-02)	Human
		AVFDII	1278–1283	Yes	No	Yes	Yes	8 (3.9e-01)	Human
NBR1_HUMAN	Q14596	LSFELL	561–566	No	No	Yes	Yes*	10 (2.0e-01)	Human
		EDYIII	730–735	Yes	Yes	Yes	Yes	17 (2.2e-02)	Human
**Additional LIRCPs from Birgisdottir et al. 2013**^33^									
BNIP3_HUMAN	Q12983	GSWVEL	16–21	Yes	No	Yes	Yes	19 (1.2e-02)	Human
		AEFLKV	159–164	No	No	Yes	No	10 (2.0e-01)	Human
MK15_HUMAN	Q8TD08	RVYQMI	338–343	Yes	No	Yes	Yes	10 (2.0e-01)	Human
CACO2_HUMAN	Q13137	FMWVTL	72–77	No	No	Yes	No	20 (8.4e-03)	Human
		DILVV	132–136	Yes	No	No	No	N/A	Human
C0H519_PLAF7	C0H519	NDWLLP	103–108	Yes	No	No	No	12 (1.2e-02)	*Plasmodium*
ATG34_YEAST	Q12292	KVYEKL	194–199	No	No	Yes	No	8 (3.9e-01)	Yeast
		FTWEEI	407–412	Yes	No	Yes	No	20 (8.4e-03)	Yeast
TAXB1_HUMAN	Q86VP1	DMLVV	139–143	Yes	No	No	No	N/A	Human
		ADFDIV	514–519	No	No	Yes	Yes	15 (4.2e-02)	Human
CTNB1_HUMAN	P35222	SHWPLI	502–507	Yes	No	No	No	11 (1.5e-01)	Human
**Data set from Behrends et al., 2010**^34^									
STK4_HUMAN [MM]	Q13043	EVFDVL	28–33	No	No	Yes	No	9 (2.8e-01)	Human
		GDYEFL	431–436	No	No	Yes	Yes	17 (2.2e-02)	Human
STK3_HUMAN [LM]	Q13188	EVFDVL	25–30	No	No	Yes	No	9 (2.8e-01)	Human
		GDFDFL	435–440	No	No	Yes	Yes	16 (3.0e-02)	Human
RASF5_HUMAN [MN]	Q8WWW0	-	-	N/A	N/A	N/A	N/A	N/A	Human
NEDD4_HUMAN [LL]	P46934	SEYIKL	410–415	No	No	Yes	No	13 (7.9e-02)	Human
		PGWVVL	589–594	No	No	Yes	Yes	19 (1.2e-02)	Human
		ESFEEL	1296–1301	No	Yes	Yes	No	13 (7.9e-02)	Human
A16L1_HUMAN [MM]	Q676U5	DEYDAL	164–169	No	Yes	Yes	Yes	16 (3.0e-02)	Human
TFCP2_HUMAN [LN]	Q12800	-	-	N/A	N/A	N/A	N/A	N/A	Human
SF3A1_HUMAN [LN]	Q15459	PEFEFI	148–153	No	No	Yes	No	13 (7.9e-02)	Human
FNBP1_HUMAN [MN]	Q96RU3	-	-	N/A	N/A	N/A	N/A	N/A	Human
TBC15_HUMAN [LL]	Q8TC07	AEWDMV	96–101	No	No	Yes	No	20 (8.4e-03)	Human
		PGFEVI	295–300	No	No	Yes	No	12 (1.1e-01)	Human
		FSFLDI	540–545	No	No	Yes	No	11 (1.5e-01)	Human
ANFY1_HUMAN [MN]	Q9P2R3	-	-	N/A	N/A	N/A	N/A	N/A	Human
TCPR2_HUMAN [LM]	O15040	GDYIAV	45–50	No	No	Yes	No	14 (5.7e-02)	Human
		AVFQLV	102–107	No	No	Yes	No	5 (1.0e+00)	Human
		AVFVAL	894–899	No	No	Yes	No	7 (5.3e-01)	Human
		DEWEVI	1406–1411	No	Yes	Yes	No	23 (3.2e-03)	Human
ECHA_HUMAN [LM]	P40939	AVFEDL	447–452	No	No	Yes	No	7 (5.3e-01)	Human
NIPS2_HUMAN [MM]	O75323	-	-	N/A	N/A	N/A	N/A	N/A	Human
ATG5_HUMAN [MM]	Q9H1Y0	-	-	N/A	N/A	N/A	N/A	N/A	Human
ATG7_HUMAN [MM]	O95352	SSFQSV	258–263	No	No	Yes	No	10 (2.0e-01)	Human
KPCI_HUMAN [LM]	P41743	-	-	N/A	N/A	N/A	N/A	N/A	Human
EPN4_HUMAN [LM]	Q14677	-	-	N/A	N/A	N/A	N/A	N/A	Human
ATG3_HUMAN [LL]	Q9NT62	-	-	N/A	N/A	N/A	N/A	N/A	Human
DYXC1_HUMAN [LL]	Q8WXU2	AVFLSL	16–21	No	No	Yes	No	6 (7.4e-01)	Human
		AMWETL	81–86	No	No	Yes	No	19 (1.2e-02)	Human
NEK9_HUMAN [LL]	Q8TD19	-	-	N/A	N/A	N/A	N/A	N/A	Human
UBA5_HUMAN [MM]	Q9GZZ9	SDYEKI	66–71	No	No	Yes	No	17 (2.2e-02)	Human
		FDYDKV	103–108	No	No	Yes	No	16 (3.0e-02)	Human
TBD2B_HUMAN [LM]	Q9UPU7	EEWELL	252–257	No	Yes	Yes	Yes	20 (8.4e-03)	Human
KBTB6_HUMAN [LL]	Q86V97	ESFEVL	120–125	No	Yes	Yes	No	13 (7.9e-02)	Human
IPO5_HUMAN [LN]	O00410	ETYENI	31–36	No	Yes	No	No	11 (1.5e-01)	Human
		DGWEFV	655–660	No	No	Yes	No	21 (6.1e-03)	Human
		LSWLPL	997–1002	No	No	Yes	No	16 (3.0e-02)	Human
NCOA7_HUMAN [LM]	Q8NI08	AEYDKL	185–190	No	No	Yes	No	13 (7.9e-02)	Human
		GEWEDL	308–313	No	No	Yes	No	19 (1.2e-02)	Human
		DDFVDL	414–419	No	Yes	Yes	Yes	18 (1.6e-02)	Human
		KSWEII	745–750	No	No	Yes	No	19 (1.2e-02)	Human
KAP0_HUMAN [MM]	P10644	EEFVEV	310–315	No	Yes	Yes	No	13 (7.9e-02)	Human
GYS1_HUMAN [NN]	P13807	-	-	N/A	N/A	N/A	N/A	N/A	Human
KBTB7_HUMAN [LL]	Q8WVZ9	ESFEVL	120–125	No	Yes	Yes	No	13 (7.9e-02)	Human
ATG2A_HUMAN [LM]	Q2TAZ0	PEYTEI	534–539	No	No	Yes	No	13 (7.9e-02)	Human
		EVYESI	828–833	No	No	Yes	No	9 (2.8e-01)	Human
		LEFLDV	1090–1095	No	No	Yes	No	9 (2.8e-01)	Human
FAN_HUMAN [ML]	Q92636	ESFEDL	600–605	No	Yes	Yes	No	12 (1.1e-01)	Human
		LVWDLL	869–874	No	No	Yes	No	13 (7.9e-02)	Human

Top rows contain sequence entries obtained from Alemu and colleagues^31^ used to construct the xLIR-motif and to validate both the cLIR- and xLIR-motifs. “Verified” signifies experimentally verified functional LIR-motifs. “Anchor” refers to a prediction of the ANCHOR software overlapping with a given LIR-motif in > 3 residues. Middle and bottom row blocks contain the additional sequences from the works of Birgisdottir and colleagues^33^ and Behrends and colleagues,^34^ respectively. Entries signified with (*) correspond to possibly spurious matches of the xLIR-motif which are simultaneously predicted as anchors, while entries marked with (**) were also present in the data set reported by Behrends and colleagues.^34^ For the atypical verified LIRs of CALCOCO2/NDP52 (CACO2_HUMAN) and TAX1BP1 (TAXB1_HUMAN), which are pentapeptides, a gapless PSSM match is not possible, thus the respective PSSM scores are marked as “N/A”. In square brackets the 2 characters correspond to one of the 3 possible observations of LIR-dependent interactions against GABARAP and MAP1LC3B, respectively, as reported in Behrends and colleagues;^34^ N, no binding against the wild-type Atg8 homolog; M, binding maintained; L, binding lost for the mutant forms.

In addition, a set of 34 human sequences tested by Behrends and colleagues^34^ for LIR-dependent interactions with GABARAP and MAP1LC3B were also retrieved from UniProt for further validating the proposed computational tools. For this purpose, UniProt was queried for human entries with the respective gene names; in a few cases where multiple UniProt entries fulfilled the search criteria we preferentially selected complete entries (as opposed to fragments) and/or entries having undergone manual curation (UniProt/SwissProt entries).

### 2.2 Generation of an xLIR-PSSM

Regular expressions (such as the xLIR-motif), albeit being useful tools for quickly scanning large volumes of sequence data for meaningful patterns, often suffer from low expressive power. One main cause for such a pitfall is that regular expressions are deterministic: a sequence either matches the pattern or not. In order to make the pattern more sensitive, an increasing number of positions become saturated, practically allowing almost all possible alternatives.

A possible workaround is to use probabilistic methods. In addition to the presence of different amino acid residues in a specific position of the pattern, such methods are able to capture the importance of each residue type occupying this position. A popular technique of this type is based on constructing a position-specific scoring matrix using as input a multiple sequence alignment. In practice, a PSSM is an L × 20 scoring matrix (where L denotes the length of the alignment), each of its elements representing a log-odds score for the presence of a residue in the respective position. Negative scores are assigned to residues rarely (even never) observed in the respective alignment column, whereas the most frequent residues are assigned high positive scores.

We used the standalone version of PSI-BLAST^35^ to construct a PSSM using the alignment of the 27 experimentally verified LIR-motifs as input and default parameters. A custom software tool was built to use this PSSM for scanning protein sequences. Since the vast majority of the known LIR-motifs are of length 6, we implement our search procedure by sliding the PSSM along the query sequence without allowing for gaps.

### 2.3 Web server development

The iLIR web server was built using the Common Gateway Interface paradigm: a user sends requests with their data to the server via a web browser (currently all major web browsers are supported). The server processes the data and sends back the results to the user's web browser for display. A limited usage of Web 2.0 technologies (i.e., JavaScript/AJAX and CSS functionalities) enables some interactivity on the results page, for example, sorting entries within the tables of sequence features or displaying tips for individual domains in the respective graphic.

### 2.4 Assessing the quality of predictions

For assessing the different predictive schemes, we resort to the simple assumption that any LIR-like motif with no experimental validation in the literature is considered a false positive (FP) when predicted to be a functional LIR. In a similar fashion, any LIR-like motif with experimental validation is considered a true positive (TP). As negative cases, we consider LIR-like motifs predicted not to be functional: false negatives (FN) when experimental evidence indicates a functional LIR-motif and true negatives (TN) otherwise. With these 4 figures available (TP, FP, TN, FN) we are able to calculate the following measures:

Overall accuracy or Accuracy (%) = 100 × (TP + TN) / (TP + FP + TN + FN)

Sensitivity (%) = 100 × TP / (TP + FN)

Specificity (%) = 100 × TN / (FP + TN)

Balanced accuracy (%) = 0.5 × (Sensitivity + Specificity)

## 3. Results

### 3.1 Sequence features of functional LIR-motifs

Previous work has highlighted the importance of specific residues located within the LIR-motif for interaction of LIRCPs with Atg8 homologs based on structural data.^29^^,^^30^ Moreover, systematic screening has led to the definition of the cLIR-motif.^31^ However, it is evident that the cLIR-motif cannot be employed to effectively detect instances of functional LIRs (thus guiding the discovery of novel LIRCPs in large scale) since only 11 out of the 27^‡^ experimentally determined LIRs are identified by the cLIR-motif (40.7% sensitivity).

To overcome this limitation, we redefined the regular expression that describes the LIR-motif in order to fit all the sequences presented in Alemu et al.^31^ Along these lines, we collected all relevant sequences from the UniProt Knowledgebase,^36^ and verified/corrected the reported position of the LIR-motifs in order to match these particular entries (cf. Table 1). We decided to keep 6 residue positions for the LIR-motifs with the conserved W/F/Y residues at position 3, in line with the definition of the cLIR-motif.^31^ After manual multiple sequence alignment of the LIR-motifs, a custom software tool produced a regular expression with all permitted residues at a given position in the multiple alignment. The resulting regular expression is [ADEFGLPRSK][DEGMSTV][**WFY**][DEILQTV][ADEFHIKLMPSTV][**ILV**], corresponding to the xLIR-motif.

### 3.2 Predictive power of LIR-motifs and anchors

The xLIR-motif matches by design all 27 experimentally verified LIR-motifs (100% sensitivity). Positions 1, 2, and 4 are less constrained compared with the cLIR-motif, whereas position 5 is more restricted. Using the background frequencies for amino acid residues in a recent version of the UniProt/SwissProt database (Table 2) we estimate the probability of occurrence of the cLIR- and xLIR-motif in random sequences drawn from this distribution as 1.8 × 10^−6^ and 1.5 × 10^−3^ respectively.^37^ This means that, overall, the xLIR-motif should be more sensitive—but less specific—compared with cLIR. In fact, this is the case since (in the same sequence data set) the xLIR-motif detects an additional set of 20 subsequences, which may be regarded as false positives (i.e., they are not functional as LIRs). As expected, the higher sensitivity of the xLIR-motif comes at the expense of lower specificity. While the overall accuracy of the cLIR-motif is somewhat higher compared with xLIR (61.7% vs. 57.4%; Table 3) this figure may be misleading due to the imbalanced nature of the data set. However, the design of the negative data set, that is, new motif instances complying with the xLIR-motif, does not permit us to compute meaningful values for specificity and balanced accuracy for the xLIR-motif (specificity 0.0% compared with 90.0% for cLIR and balanced accuracies 50% and 65.4%, respectively) (Table 3). For obtaining a more unbiased estimate of the false positive rate for these motifs, we used a sequence data set of randomized (shuffled) versions of the 27 validated LIRCPs. When scanning these sequences with the xLIR- and cLIR-motif 23 and 8 matches were reported, respectively. It is worth mentioning that this figure for the xLIR-motif is quite similar to the extra motifs identified in the original data set (20 matches). For the cLIR, this figure deviates significantly from the false positive motifs in the unshuffled sequences (2 matches).

**Table:** **Table 2.** Amino acid residue background distribution

Residue type	% Abundance	Residue type	% Abundance
Ala	8.25	Leu	9.66
Arg	5.53	Lys	5.84
Asn	4.06	Met	2.42
Asp	5.45	Phe	3.86
Cys	1.37	Pro	4.70
Gln	3.93	Ser	6.56
Glu	6.75	Thr	5.34
Gly	7.07	Trp	1.08
His	2.27	Tyr	2.92
Ile	5.96	Val	6.87

Data regarding the 20 common amino acid residues, calculated from UniProtKB/Swiss-Prot release 2013_04, April 2013; available from the ProtScale tool http://web.expasy.org/protscale.

**Table:** **Table 3.** Validation of xLIR- and cLIR-motif-based predictors

	xLIR	cLIR	xLIR + A	cLIR + A	xLIR + A + P13	xLIR + A|P13
**TP**	27	11	17	8	15	26
**TN**	0	18	16	19	18	11
**FP**	20	2	4	1	2	9
**FN**	0	16	10	19	12	1
**ACC (%)**	57.4	61.7	70.2	57.4	70.2	**78.7**
**Sens (%)**	**100.0**	40.7	63.0	29.6	55.6	96.3
**Spec (%)**	0.0	90.0	80.0	**95.0**	90.0	55.0
**BACC (%)**	50.0	65.4	71.5	62.3	72.8	**75.7**

Different schemes are validated for the prediction of functional LIR-motifs on the set of 26 proteins with validated LIRs described by Alemu and colleagues.^31^ xLIR and cLIR are based simply on the detection of the xLIR- and cLIR-motifs, respectively, whereas xLIR+A/cLIR+A require that a functional motif should overlap with an anchor as predicted by the ANCHOR tool. The 2 rightmost columns correspond to xLIR-motifs that overlap with an anchor and have a PSSM score > 13 (xLIR + A + P13) and xLIR-motifs that either overlap with an anchor or have a PSSM score > 13 (xLIR + A|P13). ACC, accuracy (%); Sens, sensitivity (%); Spec, specificity (%); BACC, balanced accuracy (%). For each validation metric the highest recorded value is depicted in bold typeface.

In the majority of the currently documented cases, the conformation of the LIR-motif is extended when bound to the LIR docking site (LDS) of Atg8 homologs. An intriguing case is the CLTC LIR-motif, which adopts an α-helical structure.^38^ If we assume that during its interaction with the LDS a LIR-motif must have an extended conformation, then it would be possible that LIR-motifs may have the characteristics of so-called “chameleon sequences”^39^ or “conformational switches,”^40^ that is, short sequences found to adopt more than one distinct secondary structure state. Such sequences have been long known to be important in protein aggregation and amyloid formation.^41^

Additionally, it has been postulated that the function of LIR-motifs may be facilitated by short-range (with respect to the LIR-motif) conformational changes. Such structural rearrangements could bring this short linear motif in a suitable extended conformation in order to interact with the 2 well-conserved hydrophobic pockets on the surface of Atg8 homologs.^29^^,^^30^ Combined with the recent observation that autophagy-related proteins are relatively rich in intrinsically disordered regions,^42^ it is possible that the LIR-motifs may adopt the required conformation after switching from a disordered to an ordered state. In order to test this hypothesis, we scanned the proteins containing experimentally verified LIR-motifs for the presence of intrinsically disordered regions using the ANCHOR software.^43^ In particular, ANCHOR predicts subsequences flanking or overlapping intrinsically disordered regions (hereinafter called 'anchors') with a high potential to be stabilized upon binding to a target molecule. Interestingly, 17 out of the 27 verified LIR-motifs (63.0%) are predicted to substantially overlap (> 3 residues) with an anchor segment (Table 1; Table 3). Even though it is difficult to draw a significant conclusion from such a small data set, it is worth mentioning that 14 out of 21 verified LIR-motifs from human LIRCPs (66.7%) overlap with an anchor, while these figures for the remaining species (namely, *Saccharomyces cerevisiae*, *Drosophila melanogaster*, *Arabidopsis thaliana*) are slightly lower (3 out of 6, or 50%). Nevertheless, it seems that the simultaneous detection of an anchor segment and a LIR-motif may be a good approach for discriminating genuine (i.e., functional) LIR-motifs. When using the cLIR-motif and posing the additional requirement that a functional LIR-motif should overlap with an anchor segment, only 8 functional LIR-motifs would be predicted as such (Table 1; Table 3), resulting in very low coverage (8/27 or 29.6%). On the other hand, the xLIR-motif in combination with anchor detection recovers 17 out of the 27 verified LIR-motifs (63.0%), at the same time eliminating most of the false positives. In fact, only 4 unverified xLIRs from human LIRCPs (Table 1) would be predicted to be functional LIR-motifs based on this compound criterion. For this scheme the figures for the xLIR-motif (cLIR-motif) combined with an overlapping anchor are: Accuracy = 70.2% (57.4%), Sensitivity = 63.0% (29.6%), Specificity = 80.0% (95%), Balanced accuracy = 71.5% (62.3%). These results clearly indicate that such a prediction scheme is far better than random.

We performed anchor predictions on the randomized versions of the known LIRCPs, and only 6 cases (out of 23) were overlapping with an xLIR-motif, which is comparable with the original sequences (4 overlapping instances of anchors with the 20 unverified xLIRs).

### 3.3 Using profile-based methods to identify functional LIR-motifs

Using the PSSM derived from the 27 experimentally verified LIR-motifs we scanned the sequences of the 26 verified LIRCPs in order to clarify whether the PSSM can be used as a more successful means to identify functional LIR-motifs. On top of the 47 hexapeptides matching the xLIR-motif (27 verified plus 20 unverified) we also obtained a score against the PSSM for a total of 18,018 hexapeptides (termed background). More specifically, by “sliding” the PSSM over each sequence one residue at a time, a score for the comparison of the PSSM to the hexapeptide starting at the given sequence position is computed. The median of scores for the 3 classes of hexapeptides (i.e., verified LIRs, unverified LIRs, background) was 18, 12, and -8, respectively, and the score distributions indicate significant differences between these classes (Fig. 1).

**Figure:**
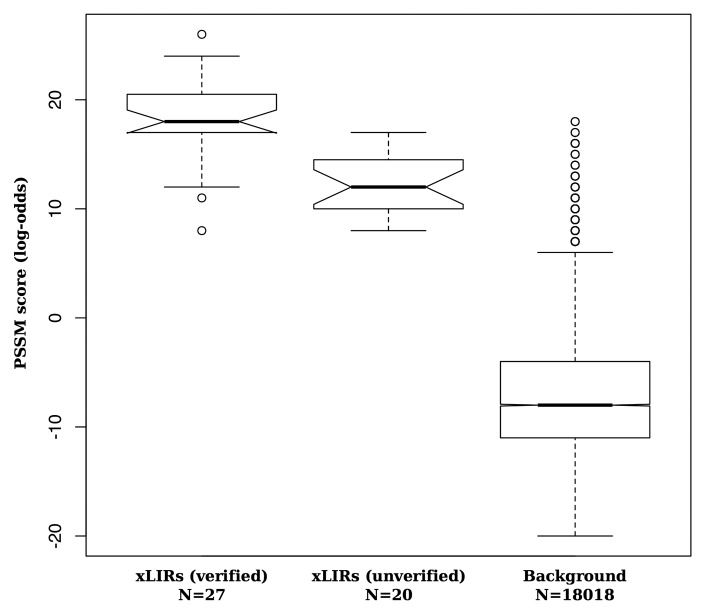
**Figure 1.** PSSM score distributions for different classes of hexapeptides. Box-plot representation of PSSM score distributions for xLIR-motifs in the 26 sequences of LIRCPs (verified and unverified; left and middle, respectively) and the remaining hexapeptides (“background”; right), obtained by scoring a sliding-PSSM along the sequences in the set of 26 sequences reported by Alemu and colleagues.^31^ The differences indicated here suggest that PSSMs may be able to reliably discriminate between functional and nonfunctional xLIRs. In particular, a Wilcoxon rank sum test with continuity correction, demonstrates significant differences between both verified and unverified xLIRs compared with background (*P* < 2.2 × 10^−16^ and 1.2 × 10^−14^, respectively) and verified vs. unverified xLIRs (*P*: 6.0 × 10^−6^).

Assuming that genuine LIR-motifs should score higher against the PSSM, we can construct PSSM-based predictors of the desired specificity/sensitivity by varying a cutoff-value for the PSSM score. We tested the power of the PSSM initially on the 27 xLIR-motifs. By trying different cutoff-values for the PSSM score (Table 4), we empirically conclude that meaningful values for this threshold lie in the range 13–17, which—when applied to all hexapeptide instances matching the xLIR-motif—give predictors of balanced accuracy in the range 74.5–83.9% with sensitivity and specificity in the ranges 59.3–88.9% and 60.0–100.0%, respectively. In terms of balanced accuracy the best performance is obtained for a PSSM score threshold of 16 (Balanced accuracy: 83.9%; Sensitivity: 77.8%; Specificity: 90.0%). However, for building a predictor solely based on this PSSM, a small (but not negligible) number of false positive hits would arise from “background” sequences (Table 4).

**Table:** **Table 4.** Validation of the PSSM method as a predictor of LIR-motifs

	Above cutoff			PSSM validation			
**PSSM score cutoff**	**xLIR (verified)** **N = 27**	**xLIR (unverified)** **N = 20**	**Background** **N = 18018** **(randomized, N = 18065)**	**ACC**	**Sens**	**Spec**	**BACC**
9	26	19	93 (85)	57.4	**96.3**	5.0	50.7
10	26	14	63 (63)	68.1	**96.3**	30.0	63.2
11	25	11	47 (49)	72.3	92.6	45.0	68.8
12	24	9	28 (32)	74.5	88.9	55.0	72.0
13	24	8	17 (25)	76.6	88.9	60.0	74.5
14	23	5	13 (16)	80.9	85.2	75.0	80.1
15	22	3	10 (14)	**83.0**	81.5	85.0	83.3
16	21	2	4 (11)	**83.0**	77.8	90.0	**83.9**
17	16	0	2 (7)	76.6	59.3	**100.0**	79.7
18	13	0	0 (5)	70.2	48.2	**100.0**	74.1

We report the number of hexapeptides with a PSSM score above different threshold values. Peptides from the background data set scoring above the threshold would be regarded as false positives if there were no restriction to comply with the xLIR-motif. Results for the randomized versions of the 26 verified LIRCPs are displayed in parentheses next to “background” data. ACC, accuracy (%); Sens, sensitivity (%); Spec, specificity (%); BACC, balanced accuracy (%). For each validation metric the highest recorded value is depicted in bold typeface.

### 3.4 Validating xLIR, anchors and PSSM with independent data sets

When this manuscript was in the final draft stage, a more complete data set of experimentally verified LIRCPs appeared in the literature.^33^ In addition to the 26 LIRCPs used for designing the xLIR-motif and validating its performance, another seven LIRCPs with an equal number of experimentally determined LIR-motifs were listed (Table 1, middle). We performed the same analyses for these 7 proteins, in order to obtain a more unbiased estimate of the performance of our approach. Interestingly, the cLIR-motif would not match any of these sequences, and xLIR would match 3 out of 7, giving 4 additional “hits.” It is worth mentioning here that the 4 missed experimentally verified LIRs include:

• human proteins CALCOCO2/NDP52, and TAX1BP1 mentioned to contain noncanonical (pentapeptide) LIR-motifs.^44^^,^^45^ It is worth mentioning that the hexapeptides starting one residue N-terminally located relative to the verified LIR-motifs yield low, yet positive, PSSM scores (7 and 8, respectively).

• the *Plasmodium falciparum* Atg3 homolog (PfAtg3), with 2 mismatches to the xLIR-motif (an asparagine and proline residue occupying positions 1 and 6 of the motif, respectively), which is, however, the highest scoring hexapeptide of this sequence against the PSSM (score = 12), and

• human CTNNB1/β-catenin, also with 2 mismatches to the xLIR-motif (a histidine and proline residue occupying positions 2 and 4 of the motif, respectively), again the top-scoring hexapeptide against the PSSM (score = 11).

Notably, none of the aforementioned LIR-motifs overlaps with a predicted anchor segment.

Another important source of LIRCP-related information stems from the work of Behrends and colleagues, in their effort for deciphering the selective autophagy protein-protein interaction network.^34^ In particular, we focus on the data presented therein to unravel the LIR-dependence of interactions of human Atg8 homologs GABARAP and MAP1LC3B with 34 proteins (Table 1, bottom). Briefly, these authors recorded binding of these 34 proteins against the wild-type and mutated forms of the Atg8 homologs (Y49A, L50A for GABARAP and F52A, L53A for MAP1LC3B). Since the residues mutated lie in the LDS and are considered critical for typical LIR-mediated interactions, maintenance of the interaction after mutation indicates LIR-independent binding, whereas loss of interaction suggests LIR-dependence. Below we summarize the computational results on those proteins that show consistent interaction patterns against both GABARAP and MAP1LC3B.

For 7 of the 9 proteins that demonstrated LIR-independence for both Atg8 homologs (marked as [MM] in Table 1) there was at least one match of the xLIR-motif (only 3 for cLIR); interestingly, only 2 of these proteins (FYCO1, FYVE and coiled-coil domain containing 1 [FYCO1_HUMAN] and ATG16L1, autophagy related 16-like 1 [*S. cerevisiae*] [A16L1_HUMAN]) had at least one xLIR overlapping with a predicted anchor.

Another 8 proteins were shown to interact with both Atg8 homologs in a LIR-dependent manner (marked as [LL] in Table 1). Six were detected to have at least an instance of the xLIR-motif (3 with cLIR) of which only 2 overlapped with an anchor: these are the validated LIR-motif of SQSTM1 and the second xLIR match of the E3 ubiquitin-protein ligase NEDD4 (PGWVVL with a PSSM score = 19). An interesting case is the serine/threonine-protein kinase NEK9, which is predicted to have 10 anchor segments, 2 of which overlap with hexapeptides scoring high against the PSSM, albeit the fact that they do not match the xLIR-motif; RGWHTI (positions: 716–721; PSSM score: 19) and DSWCLL (positions: 965–970; PSSM score: 16). Both of these hexapeptides have a single mismatch to the xLIR-motif (a His and Cys residue, respectively, at position 4) and, along with NEDD4, they could be good candidates for further experimental validation. Intriguingly, from all the known LIRCPs with a verified LIR-motif the only protein belonging to this class is SQSTM1.

Interestingly, the single case in this data set of a protein not interacting with the wild-type Atg8 homologs (GYS1) does not match either the xLIR- or the cLIR-motif.

### 3.5 The iLIR web resource

Based on the above results, we have developed the iLIR web server (identify LIR), offering a simple interface to protein sequence analysis tools, in order to facilitate discovery of novel LIRCPs. The iLIR web server is freely available to the research community at the URL http://repeat.biol.ucy.ac.cy/iLIR/; its functionalities are briefly described in the following sections.

### 3.6 Sequence input and processing

A user-submitted sequence in FASTA format is required for input (Fig. 2); it is scanned for the presence of one or more instances of the xLIR-motif. Whenever a successful hit is recorded, the matched hexapeptide is scored against the position-specific scoring matrix developed based on the experimentally verified LIR-motifs. The PSSM score is accompanied by an e-value, which represents the number of random (i.e., unrelated) hexapeptides expected to achieve a score at least as high as the one reported by chance alone. A remote BLASTP^35^ query is issued against the Protein Data Bank^46^ thus facilitating access to relevant structural data. More specifically, all significant hits with alignments including the reported motif are compiled in a list, linking to the respective PDB entry, and the complete output is also available for further analysis.

**Figure:**
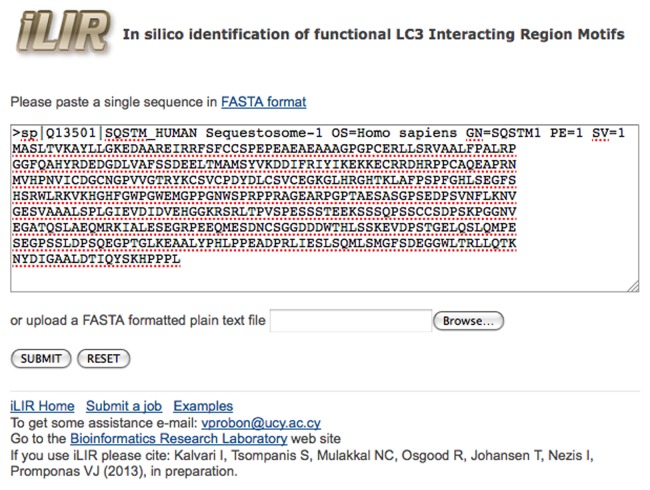
**Figure 2.** iLIR server user interface. A simple user interface enables sequence data entry in FASTA format either by copying-pasting data in the respective text area or by uploading a FASTA formatted text file. Currently, a single sequence can be processed at a given execution and no user-defined parameters are necessary/supported. Pre-run examples of known LIRCP sequences help users get accustomed to the iLIR output format.

Additionally, an automatic sequence-based query is performed against the SMART database,^47^ resulting in a list of annotated domains and motifs, including PFAM domains.^48^ Finally, the sequence is submitted to a locally installed instance of the ANCHOR package,^43^ for prediction of anchors, that is, regions within or neighboring unstructured regions with the potential to bind (following a suitable conformational change) to a globular protein.

### 3.7 iLIR output

Output results are depicted in 2 formats: a graphical depiction of detected domain motifs (similar to entries within the PFAM database) and a series of tables providing detailed information (Fig. 3; top and bottom, respectively). With regard to the graphic representation, domains are rendered using the same code utilized by the PFAM database. Moreover, generic PFAM domains are presented in orange, while domains associated with specific classes of SARs are illustrated in green. Other sequence features reported by SMART/PFAM (such as, for example, low complexity regions—blue boxes) are displayed along the sequence, with detected xLIR-motifs painted in magenta.

**Figure:**
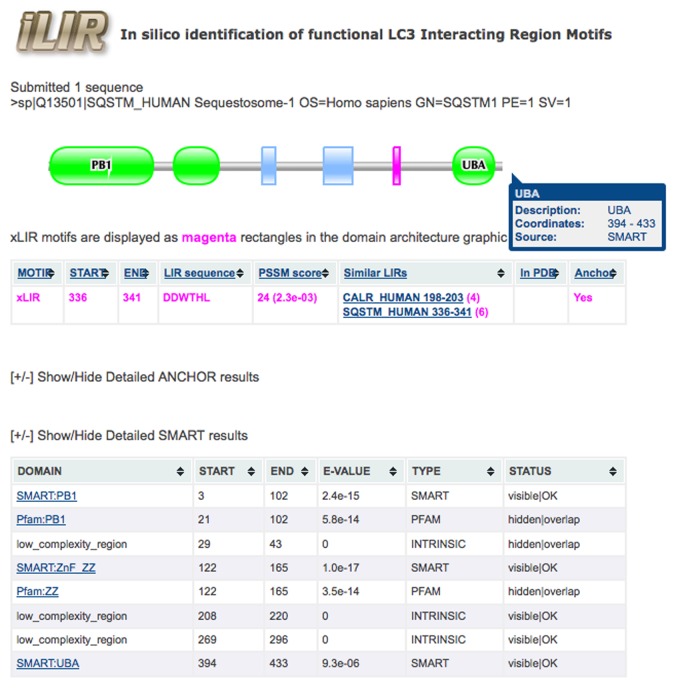
**Figure 3.** iLIR results page. The output page for human SQSTM1 (UniProt Accession: Q13501) is displayed. A graphical depiction of identified domains (top) is accompanied with detailed tables (bottom). Some domains/features are kept hidden for maintaining an uncluttered graphic but are present in the tables. By moving the mouse over any domain/feature on the graphic, a pop-up tip (blue panel, top right) displays further information (in this case, details regarding the name, borders and origin database of the ubiquitin associated [UBA] domain). Notice that the tables containing anchors and SMART-derived domains may be shown/hidden according to the user’s preference.

## 4. Discussion

We described a first approach toward elucidating sequence features that may be used to identify novel functional LIRCPs. In our work, we came up with a relaxed definition of the LIR-motif (extended LIR-motif or xLIR), which is sensitive enough for detecting a large fraction of known functional LIRs, but with reduced specificity. Even though the xLIR-motif performs substantially better compared with an earlier definition (cLIR-motif) our analysis suggests that this regular expression pattern may not be suitable for scanning large data sets (e.g., complete genomes) due to an unacceptable fraction of expected false positives. By exploiting the notion that functional LIR-motifs may transit from a disordered to an ordered state when/for binding an Atg8 homolog, we observe that more specific predictions may be achieved when restricting positive predictions in cases where an xLIR-motif overlaps with an anchor region as predicted with the ANCHOR software package. It is important to mention here that no proper data set for benchmarking predictors of functional LIR-motifs is currently available; however, our results clearly indicate that these features can be quite successful in discovering putative functional LIRs and thus detecting novel functional LIRCPs based on protein sequence information.

The rather atypical cases of LIRCPs reported recently^33^ suggest that we should expect more surprises as more knowledge accumulates in the public literature with regard to LIRCPs and their mode(s) of activity. Therefore, in order to develop more successful LIRCP predictions we may need i) to recruit orthogonal data types (e.g., evolutionary information or predicted 3-dimensional structures followed by docking) and/or ii) employ more sophisticated ways of representing LIR-motifs (e.g., profile hidden Markov models, capable of capturing dependencies between neighboring positions of the motif which are by definition neglected by the regular expression patterns and the PSSM). More experimental evidence with regard to preferential binding to particular Atg8 homologs (as for instance the work by Behrends and colleagues)^34^ or co-evolution data (e.g., concerted mutations) may unravel those molecular recognition features that are important for the LIRCP::Atg8 homolog interaction.

We developed the iLIR web server, purposely designed to guide autophagy researchers to make rational decisions on which targets to follow, rather than providing explicit predictions of putative LIR-motifs. We think that a larger quantity of high quality experimental data will be necessary in order to more accurately estimate the error rate of any such predictor.

The iLIR web server is the first tool of its kind, targeted for assisting the identification of LIRCPs, made freely available to the autophagy research community through http://repeat.biol.ucy.ac.cy/iLIR. We are actively working on examining other sequence-derived features, such as local compositional bias, predicted long intrinsic disorder, solvent accessibility, posttranslational modifications, and aggregation potential for being able to develop more successful tools for LIRCP identification.

## Abbreviations:

AIM: Atg8-family interacting motif
Atg: autophagy-related
cLIR: canonical LIR-motif
FN: false negative
FP: false positive
GABARAP: gamma-aminobutyric acid receptor associated protein
LDS: LIR docking site
LIR: LC3-interacting region
LIRCP: LIR-containing protein
MAP1LC3/LC3: microtubule-associated protein 1 light chain 3
PSSM: position-specific scoring matrix
SAR: selective autophagy receptor
SMART: Simple Modular Architecture Research Tool
SQSTM1: sequestosome 1
TN: true negative
TP: true positive
xLIR: extended LIR-motif

## Notes

Footnote

^‡^In the paper by Alemu and colleagues^31^ the most N-terminal functional LIR-motif in human TBC1D5^49^ was omitted.
